# FAIR data for optical tweezers experiments

**DOI:** 10.1016/j.bpj.2025.03.005

**Published:** 2025-03-12

**Authors:** Matthew T.J. Halma, Sowmiyaa Kumar, Jan van Eck, Sanne Abeln, Alexander Gates, Gijs J.L. Wuite

**Affiliations:** 1 Department of Physics and Astronomy, Vrije Universiteit Amsterdam, Amsterdam, North Holland, the Netherlands; 2 Lumicks B.V., Amsterdam, North Holland, the Netherlands; 3 Department of Computer Science, Vrije Universiteit, Amsterdam, North Holland, the Netherlands; 4 School of Data Science, University of Virginia, Charlottesville, Virginia

## Abstract

The single-molecule biophysics community has delivered significant impacts to our understanding of fundamental biological processes, yet the field is also siloed and has fragmented data structures, which impede data sharing and limit the ability to conduct comprehensive meta-analyses. To advance the field of optical tweezers in single-molecule biophysics, it is important that the field adopts open and collaborative data sharing that facilitate meta-analyses that combine diverse resources and supports more advanced analyses, akin to those seen in projects such as the Protein Data Bank and the 1000 Genomes Project. Here, we assess the state of data findability, accessibility, interoperability, and reusability (the FAIR principles) within the single-molecule optical tweezers field. By combining a qualitative review with quantitative tools from bibliometrics, our analysis suggests that the field has significant room for improvement in terms of FAIR adherence. Finally, we discuss the potential of compulsory data deposition and a minimal set of metadata standards to ensure reproducibility and interoperability between systems. While implementing these measures may not be straightforward, they are key steps that will enhance the integration of optical tweezers biophysics with the broader biomedical literature.

## Significance

This work defines a gap in field maturity for optical tweezers biophysics, and establishes the degree to which the field falls short of the FAIR principles. This article is significant in mapping a data infrastructure for optical tweezers moving forward, and establishes common metadata standards to be built upon. Precedents exist for fields becoming open and FAIR in the adjacent biosciences, and following their example can potentially yield great benefits for optical tweezers.

## Introduction

The increasing importance of collaboration and open knowledge sharing for scientific discovery motivates initiatives to accelerate the pace at which collaborations occur. One trend emerging over at least the past century is the “era of networked science,” which is an umbrella term encompassing several trends, including the increasing size and sophistication of collaborations, the increasingly international flavor of science, and the increase in the number of authors on an average paper over time (1). As such, collaborative skills are poised to be highly useful for the careers of emerging scientists. At the level of scientific fields, the ability to foster cooperation through governance, including data governance, such as defined by the findability, accessibility, interoperability, and reusability (FAIR) principles, will become increasingly important for maintaining active and healthy research communities. In addition, the proliferation of machine learning models increases the demand for large standardized data sets with clear structure that extends also beyond the recommendations of FAIR principles (2).

In recent decades, the rapid growth of data volumes has presented significant challenges in terms of manageability and accessibility to the scientific community. This explosion in the quantity of data affects many fields, including the natural sciences, which has transformed due to the convergence of improved computing power and the availability and affordability of genetic sequencing. Notably, scientific progress is increasingly making use of well-organized large data sets for analysis often with the help of machine learning technology. For example, the Protein Data Bank (3), UniProt (4), and GenBank (5) are well-curated repositories to store information about proteins and genes. These structured data sets have played a pivotal role in the development of powerful AI models, exemplified by AlphaFold2.0 (6). In addition, the broader ecosystem of open biological data has fostered several smaller databases for more niche research topics, such as the frameshift database (FSDB) (7), a repository of RNA sequences causing programmed ribosomal frameshifting. Whether large or small, these repositories play an important role in facilitating rapid advancements in the life sciences by ensuring proper data management.

The FAIR principles, established in a seminal 2016 publication (8), guide the FAIR of data resources for research (Table S1). Findability and accessibility are important because people can only make use of data resources that they can find and use. Interoperability is important for integration with other data types, workflows, and applications. Reusability is the main goal of the FAIR principles and can be achieved through metadata inclusion enabling replication. The development since then has gone into scoring the FAIRness of articles and data resources through various criteria (8), and automated or semi-automated workflows for FAIR scoring have emerged (9,10,11).

Historically, fields developed their own data standards. Salient examples include FASTA format for genomic data on resources such as GenBank, the Protein Data Bank (PDB) format .pdb, and the file formats associated with molecular structure data (mol2, SMILES, etc.). The PDB format was developed in 1976 and has been revised several times (current version 3.30 as of February 13, 2023). Community action was essential to establish data deposition not only as a norm, but required for publication (12) as enforced by journals and grant agencies (13). Understanding the historical development of data standards in prominent fields is valuable for shaping the future of open data resources (Table 1) (14).

**Table 1:** Information on common biological data resources

Field	Database	Data format	Organization	Founding date	Entries	Budget	Impact (no. of citations)
Structural biology	Protein Databank	PDB	Research Collaboratory for Structural Bioinformatics (RCSB)	1976	∼200k structures	∼$5 million USD/annum^a^	PDB IDs used in 585,903 publications (2021) (15)
Structural biology	AlphaFold	PDB	DeepMind	2020	>200 million entries	total expenses for DeepMind **£**595 million (16). AlphaFold costs will be a fraction of total DeepMind expenses	2021 issue cited 16,124 times^b^ (6)
Genomics	GenBank	GenBank flat	International Nucleotide Sequence Database Collaboration	1982	2.6 billion sequences	unknown	resource issues (5) have been cited 25,289 times^b^
Proteomics	UniProtKB	FASTA	European Bioinformatics Institute (EMBL-EBI)	2003 (UniProt)	568,744 sequences (14 Dec. 2022)	unknown	resource issues (4) cited 32,591 times^b^
Gene expression	Gene Expression Omnibus	SOFT file	National Center for Biotechnology Information (NCBI)	2002	∼200k data series	unknown	resource issues (17) cited 26,828 times^b^

The table includes the data resource in question, the format in which it stores entries, the number of entries, and the estimated budget, as well as predictions of impact, which is operationally defined as number of citations to the initial database article in the Nucleic Acids Research database issue.

a Based on grant sources provided on RCSB PDB (https://www.rcsb.org/pages/about-us/index#three): “RCSB PDB Core Operations are funded by the National Science Foundation (DBI-1832184), the US Department of Energy (DE-SC0019749), and the National Cancer Institute, National Institute of Allergy and Infectious Diseases, and National Institute of General Medical Sciences of the National Institutes of Health under grant R01GM133198.” NSF Grant DBI-1832184 has a budget of $8.7M USD over 5 years (2019-03-01 to 2024-02-29) or $1.74M per annum. NIH Grant R01GM133198 has a budget of $16.9M USD over 5 years (2019-08-01 to 2024-07-31) or $3.38M per annum. US Department of Energy Grant DE-SC0019749 has a budget of $0.27M USD over 8 months (2020-08-01 to 2021-03-31), as obtained by Freedom of Information Act request.

b Reference numbers are as of November 8, 2023.

Similarly, genome deposition into GenBank was established by the 1996 Bermuda Conference as a standard for publication (18,19,20). While concerns have been raised over the potential for these accessible sequences to be used for harm, as in the development of bioweapons (21) and for violating privacy (22), the deposition of sequences online has created significant research opportunities (23). Ultimately, the success of the GenBank guidelines encouraged other fields to have their own versions of these conferences, such as the development of the Amsterdam Principles for Proteomic Data (24).

The impact of public data resources in biology has been significant (Table 1). Economic estimates put the yearly financial impact of data resources curated by the European Bioinformatics Institute (EMBL-EBI) on research to be £1.3 billion in 2021 (25). Similarly, the positive economic impact of the PDB is estimated to be $5.5 billion annually according to a 2017 estimate (26). By 2017, the PDB had been used in ∼90,000 issued patents or patent applications worldwide (27). This was only the beginning, as the meteoric rise of AlphaFold demonstrated, which has already been directly cited over 16,000 times (6) (accessed November 8, 2023). Several investigational drug compounds have been discovered using AlphaFold and require further validation (28,29), experimentally determined structures still have a higher success rate for predicting drug interactions with proteins (30,31). The most recent release of AlphaFold, AlphaFold3, can predict drug interactions with proteins and may revolutionize drug discovery (32).

The development of AlphaFold was enabled due to converging developments in computing power and hardware architecture (33), cloud data storage (34), improvements in structural biology throughput providing more training data (35), and development of deep learning algorithms (36). Training deep learning models requires many structures, with annotated sequence data. Thus, the impact of AlphaFold could only be achieved by using the proteins of the PDB as a training set (6), which requires that the individual structure data be FAIR.

Individual journals have also been key to promote open data policies for publication (37,38,39,40). According to a study conducted by Vines et al. (41) in 2013, journal policies that made data archiving with a data accessibility statement mandatory were more effective than any other policy type. While many journals now have open data policies, enforcing those policies has been difficult due to the inconsistencies between an editor’s and an author’s interpretation of the policies (42).

Beyond promoting transparency, reproducibility, and interdisciplinary collaboration within the research community, several studies suggest that there is also an open access (OA) citation advantage (43,44,45,46). In particular, Piwowar and co-workers (43,45) showed that third-party data reuse is the primary reason for increased visibility and citations and that there is a 25% increase in citation rates with OA data than non-OA data. Data reuse also enables more efficient and effective progress in the research field (43,45). Another 2020 study showed an average of 25% increase in citations with articles with data accessibility statements (44). This suggests that the barrier of nonaccessibility can deter readers from using research articles.

In this study, we turn our focus toward optical tweezers (OT) data. Although the OT field has developed rapidly over the past years, with technological developments and the availability of commercial instruments driving rapid adoption, development in terms of common data infrastructure and data sharing has been limited. Furthermore, the technology possesses opportunity for integration with the broader biomedical literature and therapeutic development (47). This article uses manual and automated methods to assess the FAIRness of the field of biophysics using OT instruments.

**Figure 1:**
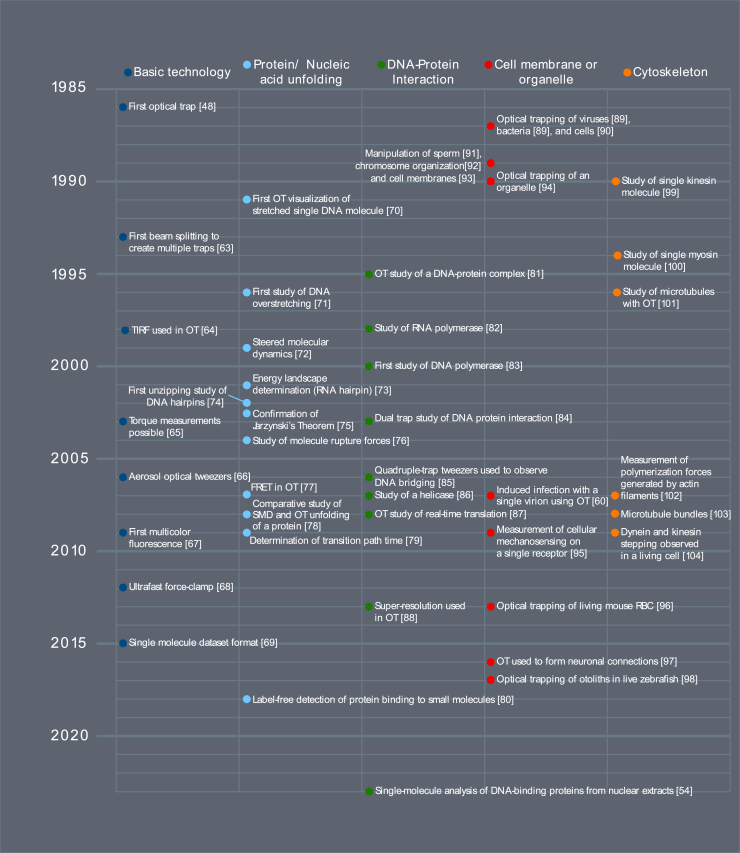
A timeline of development of optical tweezers technological capabilities and experimental systems. Some timeline items were adapted from (105). Dark blue points represent developments in the basic technology of optical tweezers (48,63,64,65,66,67,68,69), light blue points represent developments in protein and nucleic acid unfolding (70,71,72,73,74,75,76,77,78,79,80), green points denote developments in the study of DNA-protein interactions (54,81,82,83,84,85,86,87,88), red points represent important milestones in rheology of cell membranes and organelles (60,89,90,91,92,93,94,95,96,97,98), and orange points represent developments in the study of cytoskeletal components (99,100,101,102,103,104).

OT are a technology first developed in 1986 for the trapping of microscopic objects (48). OT have been used to characterize the folding pathways of biomolecules of interest, including proteins and nucleic acid structures (e.g., riboswitches) (49,50). OT experiments provide finer control over protein or nucleic acid conformation, and force can be used to induce structural transitions (47), as opposed to techniques such as FRET where one does not have the same ability to induce conformational transitions. Besides observations of the folding landscapes of protein (49) and nucleic acid structures (50), OT has also enabled the visualization of protein binding to DNA, as well as polymerase, translocase, helicase, and translation activity (Fig. 1) (51). OT assays have enabled the observation of cytoskeletal motor dynamics, even within living cells (52,53). Other capabilities have further improved the impact of OT, such as the development of multicolor fluorescence, enabling, for example, complex temporal binding dependencies (54,55). OT has also enabled the micromanipulation of cells (56), organelles (57), microswimmers (58), and viruses (59). Nanosurgery of individual cells or cell components is possible, including the experimental infection of single cells with viruses (60), and the induced connection of neurons (61).

Since their inception, OT have undergone many developments in the technological capabilities of the system as well as the sophistication of the systems being studied (Fig. 1). These have included improvements in spatial and temporal resolution (62). In addition, trap splitting has enabled users to manipulate two or more traps and to create complex experimental geometries through motion in three dimensions. Development of microfluidics has also enabled complex experimental systems to be studied, showing dependencies between multiple proteins (54). On the biochemistry side, developments in model systems, such as standardized handles for attachment, as well as technologies enabling the consistent purification, handle attachment, and fluorescent tagging of an arbitrary protein greatly expand the experimental repertoire for a researcher and improve ease of use and throughput. While a relatively new field, consistency is emerging around experimental protocols, enabling the direct comparison of different experiments. We summarize the timeline of major developments in Fig. 1, organized by: 1) developments in the basic technology (48,63,64,65,66,67,68,69), 2) protein and nucleic acid unfolding (70,71,72,73,74,75,76,77,78,79,80), 3) DNA-protein interactions (54,81,82,83,84,85,86,87,88), 4) cell membrane or organelle (60,89,90,91,92,93,94,95,96,97,98), and 5) cytoskeleton (99,100,101,102,103,104).

Despite the many technological advances, the field is still hampered by a lack of consistency in data standards, a challenge that has been identified by others in the field (106). While attempts have been made to create a common standard, there is limited adoption (107). Here, we will examine the degree to which OT data abide by the FAIR criteria through an analysis of OT articles published within the last decade. The analysis of articles published within OT shows an increasing trend, where articles having an associated data set (as opposed to none) become a greater proportion of OT articles over time. The proportion of OT articles that are OA has risen from 15% in 2010 to 45% in 2021. This trend is encouraging, although OA is not synonymous with FAIR, and a minority of the top-cited OT articles comply with FAIR standards. We also discuss the types of experiments performed on OT and using a previously described categorization. For these experiments we put forward a minimum reporting standards, which differ based on the processes studied: DNA-protein interactions, protein/nucleic acid unfolding experiments, rheology, and force generation by cytoskeletal filaments or motors. Finally, we set out basic standards for reporting OT data, analogous to the metadata requirements of the PDB. These metadata standards are sufficient and necessary for publications to meet FAIR, after which the development of a centralized repository may be a future development.

## Methods

### FAIR assessment of OT articles

#### Manual analysis

To assess the adoption of FAIR policy within the OT community, we conducted a two-phase literature review. In the first phase, we conducted an analysis examining data availability in the 10 most highly cited papers on OT published between 2015 and 2020. Using Mendeley’s database, we searched for "optical tweezers" and ranked results by citation count, and assessed if there was an attached raw data set (see Table S2). The article that included a raw data set was then assessed for adherence to the FAIR criteria included in Table S1, producing Table S3.

According to the FAIRisFAIR survey performed in 2019 (108), science publishers adopted FAIR data policies ranging from 2014 for Public Library of Science to 2018 for Taylor & Francis. We wished to assess a sample that more rigorously followed FAIR guidelines. We conducted an additional search in the OpenAlex database focusing on publications from 2019 to 2020, after major journals had implemented FAIR data policies. We specifically looked for research articles that primarily used OT in their experimental methods and were published in journals adhering to FAIR data guidelines (108). We chose the top 10 cited experimental articles using OT and assessed their FAIR adherence, first observing if they had raw data associated with the publication (Table S4). One article had raw data available (109), and one other had a link to a Dryad repository, which was not functioning as of this writing (accessed December 5, 2024) (110). The point-by-point analysis of each of the FAIR criteria is included in Table S5.

#### Automated analysis

In the second phase, we leveraged a large bibliometric database, OpenAlex (111), to quantitatively estimate the extent of FAIR data in the OT literature. We identified 5193 journal articles categorized with the *Optical Tweezers* concept (C20198109) and published between January 2010 and May 2022 in the OpenAlex database (111). We then focused on the 3137 articles published under an OA license, allowing us to download the full text pdf of the publication. All URLs were then extracted from the article full text, resulting in 1667 URLs pointing toward a data set or supplemental file from 835 articles (Table S6).

## Results

### The yearly output of OT publications is increasing

**Figure 2:**
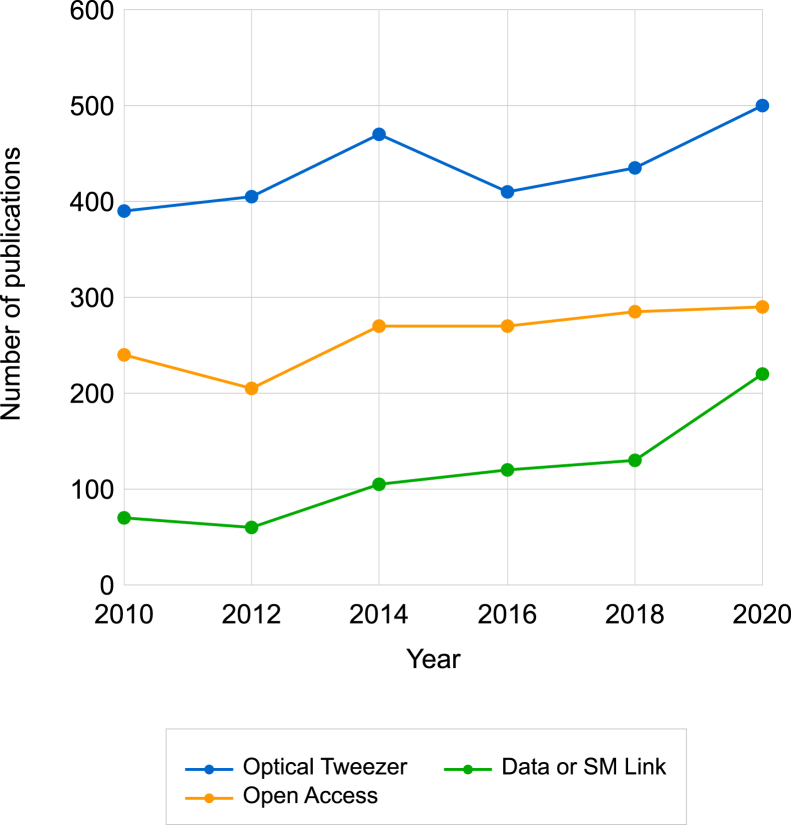
Overview of open access in optical tweezers literature. Yearly number of publications that are categorized as (*blue*) optical tweezers (OT), (*orange*) OT and open access, and (*green*) OT and the full text contained a URL to a data set or supplement resource.

A search for articles using the query “Optical Tweezers” yields the following result: the total number of publications has seen an increase in the number of publications per year over time, from ∼400 in 2010 to ∼500 in 2021 (*blue line*, Fig. 2). Of these, roughly 60% are OA (*orange line*, Fig. 2), and ∼29% have an outgoing link to a data set or supplemental materials file (*green line*, Fig. 2). Worldwide, of articles published between 2015 and 2019, OA articles represent 47% of total publications in all science fields (112), so OT has a higher proportion of OA publications than the rest of science. Next, we considered only on the OA subset of articles and computationally infer the FAIR status of the linked data sets using the F-UJI model (113). The F-UJI model identifies linked content and scores the data set based on objective criteria for the individual FAIR components. 26% of OA articles link to FAIR data resources (Fig. 2), when averaged over the period 2010–2021.

### Top articles lack FAIR datasets

**Figure 3:**
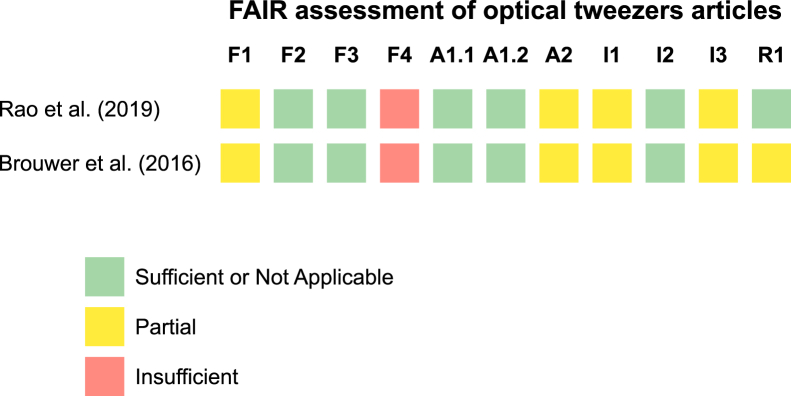
Manual analysis of two optical tweezers articles in terms of the FAIR criteria. An analysis of articles (55,109) for FAIR adherence (Table S1). Green denotes meeting criteria, yellow denotes partial meeting of criteria (such as when some data entities link to persistent identifiers for criteria F1, but not all), and red denotes failure to meet criteria. Individual criteria are given in Table S1. F1-4 refer to the “findability” criteria, whereas A1-2 refer to the “accessibility” criteria, I1-3 refer to the “interoperability” criteria, and R1.1–1.3 refer to the “reusability” criteria.

Our manual examination of the 10 most highly cited OT papers published between 2015 and 2020 revealed that only one study (55) (Fig. 3) included an accessible data set. Looking at the analyzed data availability among the 10 high-impact OT papers, the results painted a clear picture. The majority—6 papers—provided no information about their data sets. Of the remaining 4 papers, two required potential users to contact the authors directly to access the data. Only 1 paper included representative data traces within their supplemental figures, while just a single paper provided comprehensive data for all its figures. From the search of 10 papers from journals with open data policies (2019–2020), only 1 additional paper provided a usable force-extension data set (109). In addition, 1 article included a link to a Dryad repository, but the link was not functioning (110). This distribution clearly demonstrates limited data accessibility in this field.

A limitation of our methodology is that it favors high-impact publications, particularly those that are OA and more likely to be subject to stringent data sharing requirements. This likely results in our analysis overstating the actual level of FAIR compliance within the field. Moreover, OT instruments are often expensive, and they are more likely to be located in well-funded institutions with agreements to publish OA or resources to cover publication costs. This may create a possible bias in our search toward well-capitalized labs, typically in developed countries. OA publishing is more likely to come with data sharing requirements, and OA articles are more likely to show up in search results. It is possible that our search misses lower impact factor and non-OA publications, possibly overstating the level of FAIR adherence in the field.

### Classification of experimental types

We next employ a qualitative categorization on the OT articles to identify common experimental types and observables. At the most basic, there are fundamental physics works on optical trapping, which typically examine the trapped object in isolation. The next level consists of publications that analyze the mechanical properties and/or molecular functions of biomolecules. Chronologically, this corresponds to the early 1990s in terms of development of the field, and included such experiments as measuring the persistence length, stretch modulus, and stretching behavior of biopolymers (114). Chronologically afterward, the field forks: with one area that looked at specific structures (proteins, nucleic acid pseudoknots) in unfolding experiments, and the other functionality developed to examine interactions of nucleic acids with interacting proteins.

Crucially, molecular motors research served as a major catalyst for advancing OT capabilities. Pioneering studies in this area developed groundbreaking techniques such as beam splitting (63), total internal reflection fluorescence integration with OT (64), and ultrafast clamp spectroscopy (68), enabling unprecedented manipulation and measurement of cytoskeletal filaments and motor proteins (52). These methodological advances not only expanded OT’s utility in studying motor mechanics but also laid the foundation for broader applications in nucleic acid research and cellular biophysics, including studies of membrane properties (115) or cell motility (116).

The main experimental types using OT are described in a recent review (117), these are grouped by the object or phenomenon studied, including:

(1) DNA-protein interactions
(2) protein/nucleic acid unfolding experiments
(3) rheology
(4) force generation by cytoskeletal filaments or motors (“Cellular structure and transport” in the cited review)

**Figure 4:**
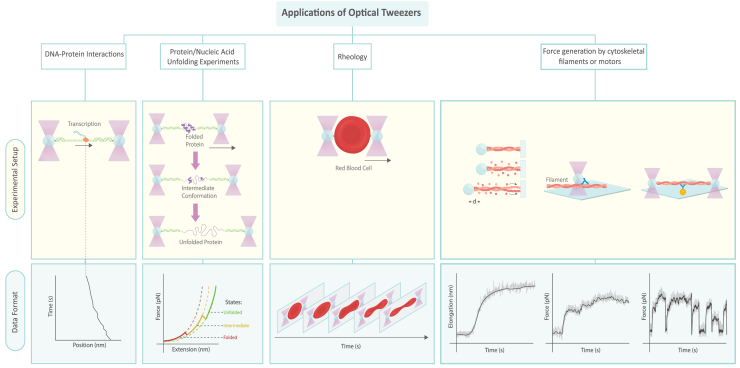
Main experimental setups and data types for optical tweezers. Tan boxes show the experimental setup, whereas shaded blue boxes show the typical data trace for the experiment. The systems covered are protein/nucleic acid unfolding experiments, which consist of repeatedly unfolding a protein or nucleic acid construct to produce a force-extension curve. DNA-protein interactions measure the dynamics of a DNA-interacting protein, typically using a fluorescently tagged protein, although information can be obtained purely from the force-extension curve. A typical data trace from this experiment, called a kymograph, shows the position of the protein as a function of time. Rheology experiments measure the dynamics of cells, producing a movie as the typical data trace. Studies of force generation by cytoskeletal filaments or motors study cytoskeletal components and associated transport processes, such as with actin filaments. Data traces from the experiments (102) (*left*) and (125) (*center* and *right*).The left setup shows a means to measure the polymerization of a cytoskeletal filament, and the data trace (*lower*) shows the growth of a filament with time (102). The center setup shows the one bead assay for the dynamics of a cytoskeletal motor. A filament is deposited on a coverslip, and a bead with a “walker” attached is able to translocate across the filament, while pulling the bead, which provides a measurement of force (125). The three-bead assay (*right*) deposits a bead with an attached motor on the coverslip, and the filament is held between two traps. Typical data traces are shown below, showing force versus time (125).

Each experimental type has an associated observable quantity as well as derived quantities (Table 2; Fig. 4). The quantities will depend on the parameters of the experiment, and it is important that the metadata are recorded. For example, the diffusion constant is derived from the position distribution of a typically nonprocessive protein. This framework is broad enough to encompass a significant proportion of OT experiments today. There are a few other uses of OT, particularly as a method for cell sorting (118) as well as studying the nanophysics of nonbiological systems (119). The reader may find general reviews on the interpretation of data for DNA-protein interactions (120), protein and nucleic acid unfolding experiments (121), rheology (122,123), and cytoskeletal motors (53,124).

**Table 2:** Experimental types, object of study, observables, and inferred information

Experiment type	Object of study/system components	Measured quantities	Inferred information
DNA-protein interactions	nucleic acid strand and interacting protein	force and extension as functions of time; positions (by fluorescence) of proteins of interest as functions of time	binding constants, diffusion, cooperativity, velocity, processivity, pause distribution, step size, step kinetics
Protein/nucleic acid unfolding experiments	single biomolecule capable of folding	force and extension as functions of time	(un)folding forces, (un)folding pathways, energies, unfolding rates, energy landscape, transition/state map, state identities (3D conformation)
Rheology	cell, organelle, polymer networks, or cytosol	morphology and applied force as functions of time	deformation energy, Young’s modulus
Force generation by cytoskeletal filaments or motors	cytoskeletal components (e.g., actin, myosin, kinesin, dynein, microtubules)	force and displacement as functions of time; positions (by fluorescence) of proteins of interest as functions of time	polymerization mode, binding constants, diffusion, cooperativity, velocity, processivity, pause distribution, step size, step kinetics, stall force

For each experimental type, there is an object of study and an experimental observable. Information inferred from analysis is included in the rightmost column.

Broadly, unfolding experiments have the force extension curve as their data type. DNA protein interactions with fluorescently labeled proteins produce the kymograph as the data set. Rheology is image based, which can be analyzed to extract information about morphology and forces.

Studies on force generation by cytoskeletal filaments or motors employ a variety of experimental techniques to analyze the dynamics and mechanics of these cellular components. While elongation versus time or force versus time traces are typically used as the primary data set, image-based data can also be incorporated when fluorescence microscopy is employed. These experiments often focus on cytoskeletal processes such as filament polymerization and motor protein dynamics. For instance, filament polymerization assays measure the growth of cytoskeletal filaments over time, producing elongation versus time traces (102). One-bead assays, used to study cytoskeletal motor dynamics, involve a filament deposited on a coverslip with a bead attached to a "walker" molecule. As the motor protein translocates along the filament, it pulls the bead, allowing for force measurements. In three-bead assays, a bead with an attached motor is fixed to a coverslip, while the filament is held between two optical traps, enabling precise force measurements as the motor interacts with the filament (125). The one-bead and three-bead assays are not equivalent (125,126,127), so care must be taken in interpreting data.

OT experiments have developed considerably with the incorporation of fluorescent imaging of proteins (128). This allows the user to observe the impact of biopolymer tension on protein mechanics, including protein binding and processivity, the average distance that a molecular motor traverses (forward) before dissociation. It is possible to obtain useful information about the system without using imaging, but imaging adds considerably to the versatility of the system. The main data output of combined force-fluorescence experiments is the kymograph, which shows the position of the fluorescent protein on the nucleic acid strand between the beads (128). Combined force-fluorescence experiments are an active field of research, and analysis has high complexity and information, especially using multicolor fluorescence and multiple proteins (54).

### Necessary reporting conditions for metadata

It is important to include metadata for experimental reproducibility and annotation of experiments. In other fields, this has enabled the creation of data sets based on metadata standards. One example from clinical medicine is standardized reporting in meta-analyses, called the Preferred Reporting Items for Systematic reviews and Meta-Analyses (PRISMA) guidelines, which allows the reader to obtain the necessary information from a systematic review (129). Other examples include the metadata standards for figures when publishing, such as the Nature Portfolio group of journals releasing their reporting guidelines, requiring that reporting be standardized and thorough in science research (130). These checklists that were released and required for publishing naturally drove up the proportion of articles with complete metadata (131).

In the case of OT, metadata should be sufficient to ensure reproducibility, and metadata fields may possibly be used as a variable in a retrospective analysis. The metadata that are necessary to include are given in Table 3, showing the quantities in the left-hand column and the units in the right-hand column. Hardware conditions should apply to all OT experiments, regardless of the object of study. Assay conditions in Table 3 are included for all experimental types. Both unfolding experiments and DNA-protein interactions use nucleic acid handles, which can be represented as an annotated FASTA sequence. Construct conditions refers to the object of study, which may be specified by a FASTA sequence or even a PDB ID, where applicable.

**Table 3:** Necessary values to include to ensure replicability of experiments

Hardware conditions	
Value	units
Make of setup	brand name or custom setup
Model number	
Spatial laser control	acoustic-optical device (AOD), electro-optical device (EOD), mirror
Detection device	quadrant photodiode (QPD), camera
Objective lens	make and model no.
	lens parameters
Optical filters	make and model no.
	filter parameters
Laser power at source	mW
Laser power at objective	mW
Trap stiffness	pN/nm
Uncertainty in trap stiffness	pN/nm
Temperature	K
Laser wavelength	nm
Acquisition rate	kHz
Line scanning rate	Hz
Filter frequency	Hz
Calibration technique	method (active and passive power-spectrum, equipartition theorem, etc.) (132)

Assay conditions				
Nucleic acid or protein unfolding experiments	DNA-protein Interactions	force generation by cytoskeletal filaments or motors		rheology
		bead geometry	polymerization assay, one-bead, or three-bead assay	
Bead size		nm		
Uncertainty in bead size		nm		
Bead functionalization		name (anti-digoxigenin, streptavidin, etc.)		
Trap control		force ramp, force clamp, constant force, constant distance, force jump, custom		
Force profile		force (pN) vs. time (s) trace		
Extension profile		extension (pN) vs. time (s) trace		
Handle sequences		FASTA nucleotide with annotations for bead attachments		
Flow pressure (if applicable)		mbar		
Linear flow speed at bead		mm/s		

Construct parameters					
Nucleic acid or protein unfolding experiments	DNA-protein interactions	force generation by cytoskeletal filaments or motors		rheology	
Sequence identity	FASTA sequence, PDB ID^a^, UniProt ID (for proteins)^a^	structure type	microfilaments (actin), intermediate filaments, or microtubules	cell identity	Cell Ontology ID (133) Human Cell Atlas ID (if human) (134)
Fluorescent labels	sequence location, identifier	protein unit(s)	Gene Symbol, FASTA sequence, PDB ID,^a^ UniProt ID^a^	cell genome	FASTA Sequence or GenBank ID
		perturbations to structure structural unit	custom field	perturbations to cell	custom field
Absorption wavelength	nm				
Emission wavelength	nm				
Quantum yield	%				
Labeling efficiency	%				

**Factors in solution**					

Identity		name			
Concentration		nM			

**Small molecule**					

Identifier		name, CAS number, structure, SMILES			
Concentration		nM			

**Protein/peptide**					

Identifier		FASTA sequence, PDB ID,^a^ UniProt ID^a^			
Fluorescent labels		sequence location, identifier			

**Nucleic acid**					

Identifier		FASTA sequence, GenBank accession,^a^ PDB ID^a^			
Fluorescent labels		sequence location, identifier			

Fields traversing multiple columns are common to those experiments that the fields span. For example, the hardware conditions, spanning the entire width of the table, are common to all experiments, whereas sequence identity is common only to unfolding experiments and DNA-protein interactions. The parameters are divided into hardware conditions, Assay conditions, construct parameters, and solution factors. Hardware conditions pertain to the parameters of the OT hardware, including laser power, trap stiffnesses, and other parameters. Assay conditions refer to properties common to the assay, including the bead sizes and handle parameters. Construct parameters refers to the properties of the experimental construct being measured, providing the construct sequence and other necessary metadata. Lastly, the factors in solution parameters enables a replicable description of experimental solutions, including buffers, small molecules, and proteins or nucleic acid molecules in solution.

a Denotes “if applicable.”

Solution factors refers to elements in solution, which may interact with the construct or impact the DNA-protein interaction. These can be small molecules, oligonucleotides, or other proteins in solution. These should be specified, along with their concentration.

### Standardization of data structure

Given that 835 of the 5193 (16.1%) journal articles on OT are associated with a data set, less still pass the FAIR standards. Ensuring the accessibility of OT experimental data may greatly improve field progress. If data are shared, this can enable researchers to enhance their analysis in many ways, such as to probe transient conformations of proteins and nucleic acid structures and integrate the results with structural studies (47), to perform meta-analysis on OT data (135), and to categorize and organize experimental conditions, including sequence (136,137), compounds (138), oligonucleotides (139,140), temperature (141,142), salt (143), and others. With a single database, it would be possible to refine workflows using machine learning, as well as create models for simulation of structural dynamics, adding another layer of information to static structural models. These could also be used as benchmarks for quality control and calibration of experimental systems.

To begin, we propose that all publications using OT data include a minimal set of metadata enabling experimental replication, and possible harmonization in cases where parameters are different. These parameters need to include the solution components and concentrations of one’s flow cell channels, as well as temperature and pH. The attributes of the machine that must be recorded are the trap stiffness, and laser wavelength and power. The bead size, handle sequence and length, and attachment geometry are part of a set of minimal reporting guidelines, as well as the sequence of the target protein or nucleic acid structure. Any ligands and other biomolecules must also be included. Beyond this, any dynamic trap parameters must also be included to ensure replicability, including force ramp rates or pulling rates and driving frequencies in the case of rheology experiments.

One major challenge is that the analysis pipelines for OT data are still expanding, and there is no explicit consensus on what a “standard” experiment constitutes. New experiments are being constantly observed and each bring a different challenge to analysis. For example, Schaich et al. (54) developed an analysis of sequential binding, which took advantage of three-color fluorescence. A novel analysis technique was developed based on the colocalization of the different color dyes and the order of their binding to observe the sequential dynamics of DNA repair proteins (54). While these analyses can be highly complex, establishing foundational automated processing tools provides essential basic functionality. This groundwork can then support the development of more sophisticated analytical methods. Nonetheless, a minimal data set should be agreed upon and is suggested below.

### AI-ready single-molecule unfolding data

AI-ready data require consistently structured data that can be used as a training set. In the case of single-molecule unfolding experiments, a structured technique of assigning OT experimental data to 3D structures is necessary for this application. For this case, AI may be used to assign individual OT branches to specific conformations, thereby elucidating the conformational dynamics of a target protein. This may be useful for drug discovery, as this may provide intimate detail into molecular structure/function relationships (47).

Parallel to the developments necessary to effective protein structure prediction, the same trends are present in the field of single-molecule OT research. Throughput is improving owing to technological developments in the field. However, meta-analysis of multiple data sets remains a challenge owing to the low FAIR adherence in the field. This situation of data siloing makes machine learning difficult. For the case of protein structure prediction, performance of models converged above training set sizes of 2000 protein chains or above (144), establishing this as an approximate minimum threshold for training set size in protein structure prediction. It is unknown what the threshold would be to build a model based on force-distance curves, and currently limited data are available.

A search of PubMed literature specifically oriented to protein unfolding experiments yields 97 suitable publications (search terms “protein unfolding optical tweezers”, excluding publication type = “Review”, accessed November 8, 2023), providing a small data set to begin. If a similar proportion of this experimental subtype has FAIR data (∼40%), this leaves us with 39 accessible data sets. A heuristic for training set size is that it should be at least 10–100 times the number of model features (145), so lack of data may be a barrier, at least at first, although more accessible data alleviate that.

Currently, we do not know how much data would be necessary to accurately predict the unfolding and folding landscape of a protein or nucleic acid structure, which can often be complex and nonintuitive (140). Protein structure prediction previously relied on molecular dynamics equilibration (146) using established force fields based on of physical principles (e.g., electrostatics) (147). Analogously, predicting the unfolding landscape of a protein is currently based on physical principles (148,149). One additional challenge is that there is no way to verify the identities of states in the case of protein unfolding; while there are useful heuristics, these transition maps are fundamentally predictions, and it remains impossible to identify with 100% certainty these transient and intermediate states. More sophisticated machine learning models may incorporate pairwise FRET distances set between two residues on a protein to constrain the solution space (146).

Other possible applications of AI in OT could focus at the cellular motility level, for example, machine learning of the response of cells to a mechanical perturbation may inform cytosolic dynamics, and the localization of cargo and organelles. AI can also be important as a tool in OT, such as automated cell sorting based on more subtle characteristics than those sorted in standard cell cytometry (150), as well as use in single-cell infection assays, which provide a clearer view into viral mechanisms and the actions of antivirals (60). The possibilities to alter cell morphology and perform nanosurgery is also ripe for automation, and this can provide very fine-grained insights into cell function (151).

### Development of a common data format for OT experiments

To move forward the OT field requires a common data format to enable interoperability (107). Fortunately, interoperable data formats already exist through the use of consumer OT hardware, which output data in a structured manner that meets the FAIR requirements. There are still several distinct formats of OT data that exist, as early work was performed on custom-built hardware, and other hardware suppliers exist in the field. At the very least, these should be able to be converted, with metadata recorded.

The essential metadata should follow the minimal reporting requirements in Table 3 and should contain the following elements.

#### Findable

A researcher looking for relevant data must first be able to find it. For this requirement to be met, data sets must be indexed by the system being studied. In the case of DNA-protein interactions experiments, the protein/gene name must be included in the data set in its own field. It should also clearly be marked by experimental methodology (in our case OT). For example, a recent OT publication (152) has a data set associated with it, which clearly denotes that it is examining the T7 gp2.5 system in the Data Set description. The experimental methodology is briefly summarized in the associated data set, as investigating “the binding dynamics of T7 gp2.5 and a deletion mutant lacking 21 C-terminal residues (gp2.5-Δ21C) under various template tensions” (152). Ideally, these entries should be linked to relevant entries in other databases: UniProtKB and PDB for proteins, CHEMBL for small molecules, gene expression omnibus for transcriptomes, and GenBank for DNA.

#### Accessible

The article presenting the data should link to a file download of the data used in the figures of the published work and be available as a link in the article body or supplemental information. In our example, the link is clearly provided as a link in the Data Availability statement to the Zenodo directory (152).

Accessibility may also refer to the accessibility of tools for interpretation and analysis of OT data. We recommend the following guides (previously mentioned) to data analysis for DNA-protein interactions (120), protein and nucleic acid unfolding experiments (121), rheology (122,123), and cytoskeletal motors (53,124). Making these toolkits available by means of code and data repositories can help to improve accessibility.

#### Interoperable

Metadata on experimental conditions must be included such that the data may be compared with other data sets with different parameters. For example, the pulling speed will influence unfolding forces. In principle, this can be adjusted for, but the pulling speed must be recorded as a metadata parameter of the experiment. Data should be in a format that enables conversion, and not a proprietary format. In our manual search of publication data sets, we found files with Excel, MatLab, or Text extensions, making it harder for a single parser to read. Compressing data into a single JSON format such as SMD would provide a better machine-readable experience. These problems can be solved by a domain-specific repository with restrictions for uploading data sets.

Conversion between formats will be a necessary functionality. Databases usually host one or a small set of formats, such as the PDB’s .pdb file format (3) (Table 1). Eventually, both commercial manufacturers and research laboratories will either adopt the prevailing data format or ensure their formats can be converted to it.

#### Reusable

Metadata must be sufficient to ensure that the experimental results are replicable. This means that metadata for force-fluorescence experiments must include all the relevant experimental conditions, including sample concentrations, trap stiffnesses, and bead parameters (Table 3). Other quantities, such as bead positions and forces, are recorded constantly throughout the experiment. The OT manufacturer LUMICKS B.V. has an internal python file format for storing whole experiments with combined force and fluorescence, given in the PyLake package (153). A recent publication has also developed their own storage and analysis package for combined force-fluorescence experiments (154).

#### AI-ready

Increasingly, FAIR data are being incorporated with the additional criteria of being AI-ready. This requires an explicit training set against a known result. The readiest applications of AI in OT is in unfolding experiments, where applications such as AlphaFold can be extended to take into account the conformational ensemble of a protein, as opposed to static states. An intermediate goal could be assigning gross structural states to force-distance curve branches. Gross structural states can be described by a secondary structure where a determination is considered likely. Development in this pipeline is necessary, including confidence estimates in state assignments.

While other applications exist, such as in cell rheology, where machine learning can be used to analyze cell deformations and eventually predict stress responses, the use case of single-molecule unfolding remains primary.

### Further recommendations

#### Make data set deposition the standard for publication

To improve the numbers of data sets available and the field transparency, going forward, data sets should be shared to enhance the collaborative ability of the field and the ability to draw insights from disparate experiments, as well as provide benchmarks. This also increases the data available to train machine learning models, which can benefit the field and be used to predict behavior without the need for experiments. Here, OT can establish themselves within the drug discovery process (47).

#### Development of a database for OT experiments

While initially, OT data sets could be deposited in a decentralized way through resources such as Figshare, Dryad, or Zenodo, field development will motivate the creation of a searchable repository of OT data, which allows public people to download and provides sufficient metadata that are interoperable and reusable. This resource will collect data and allow them to be sliced by metadata attributes to obtain more general insights into protein and nucleic acid folding and be used for the development of predictive models. A centralized resource with access to experimental data is preferred, as this allows researchers to access many different data sets with greater convenience; however, this is not explicitly necessary for FAIR adherence as long as the data exist and are accessible. Adoption of standards can predate the development of a federated server.

One aspect of open data sharing that may impede its adoption within a scientific community is the substantial resources required for the maintenance of data-sharing services. Sustaining public databases for long-term data curation and preservation of scientific data demands financial support that surpasses the funding allocated to an individual research project by approximately 16-fold (155). The cost of open data resources has typically been borne by governmental grants, which is subject to volatility (156), although the importance of open data has received significant appreciation by funding agencies in recent years, including an open data initiative by the National Institutes of Health (157).

FAIR and open data in single-molecule biophysics provides a boost to a field with much untapped potential (47). With data set sharing, significant benefits will accrue to the field, increasing possibilities for collaboration, meta-analysis, and participation by those lacking resources to purchase capital intensive instruments. These previous initiatives (Table 1) show the positive impact of community leadership toward open and FAIR data policies.

#### Improving connections with other fields and data sources

For the maturity of OT data to reach its full potential, it must link to other related data sets, which can be accomplished in the data depository. From here, independent researchers will develop bioinformatic workflows. One example may be to explore the conformational space of a protein through an analysis of unfolding and refolding curves and state matching with alternate structural conformations.

The development of bioinformatic workflows will have unanticipated positive consequences, but one immediate consequence will be the applicability of OT to drug discovery by expanding the conformational space of a given target, enabling the discovery of drugs targeting specific conformations, as well as finding drugs for undruggable protein targets (47).

We propose that journals demonstrate a commitment to innovation through open data by recommending deposition of data sets for OT for every figure; if required, data can be downsampled to decrease file sizes. To allay concerns of being “scooped,” data sets can be embargoed for an agreed-upon period of time, where they retain exclusive rights to their data, a policy that other biological databases practice (122). A collaborative environment within OT supports collaboration and innovation and will be of great benefit to those within the field, and also those downstream of its impact on the biomedical sciences.

#### Encourage data authorship

Annotating and uploading data takes time and many researchers would prefer to keep a data set to themselves to obtain multiple publications from it. In our analysis, data being available upon request to the authors was a common means of providing access to data. While this can be inconvenient, it is in principle possible to access data if a researcher submits a reasonable request. The project FirstApproval.io (158) provides a data repository, however, in which authors are incentivized to host their data by getting authorship credit on publications that use their data. This model may be an improvement on explicit requirements to include data, which are often seen as a barrier to publication.

Given that data sharing is valuable, authors may need to be incentivized to share data (159). As a field, OT often has a low throughput when compared with other experiments, and this may contribute to a greater reluctance to share. Common data formats and analysis pipelines can lower the barriers to FAIR adoption, and data coauthorship provides a potential model for incentivization.

## Conclusion

Data sharing has been revolutionary in biomedical sciences, enabling innovation and supporting collaboration. Current data resources provide significant benefit, not only to researchers but to industry and the public. While comparatively immature in this regard (data sharing), the field of OT can benefit from the precedents of other successful biological data resources. Data sharing has enabled the use of previously underutilized data resources, often used in a single publication and not reused. Latent potentials exist in broadening access to single-molecule data, and the authors expect greater interoperability with other data sources and greater interdisciplinary transfer, such as between single-molecule biophysics and structural biology or medicine. Open and FAIR data also unlocks more general insights into the experimental phenomena being studied. For example, while several unfolding experiments do perturb the system through mutations, oligonucleotides, or by titrating ions, drug-like molecules, or biological macromolecules in the solution, the analysis remains confined to the single experimental system. More generalizable insights may come from looking for patterns within more heterogeneous studies, and this becomes a possibility when data is available. Presently, the level of reuse is limited by the availability of data (Tables S2 and S4). As mentioned, most of the data requests need to be performed manually and, while authors receiving requests are held to be forthcoming by professional courtesy, requests can be laborious and easily ignored. Bringing OT data to FAIR standards is not without its challenges, and the heterogeneity between different experiments is large. The adoption of common standards has been a way to mark maturity across many fields, and OT can benefit from greater standardization and interoperability.

## Data availability

All of the data analyzed in this article are available within the supplemental files.

## Acknowledgments

This work is supported by European Union H2020 Marie-Sklowdowska Curie International Training Network AntiHelix (859853).

## Author contributions

M.T.J.H. designed research, analyzed data, and wrote and edited the manuscript. S.K. analyzed data and wrote and edited the manuscript. J.v.E. designed research, analyzed data, and wrote and edited the manuscript. S.A. designed research and edited the manuscript. A.G. designed research, analyzed data, contributed analytical tools, and wrote and edited the manuscript. G.J.L.W. administered and supervised the project and edited the manuscript.

## Declaration of interests

M.T.J.H. is a former employee and G.J.L.W. is a cofounder of LUMICKS B.V., an optical tweezers manufacturer. S.A. is in a consortium agreement with Olink and Quanterix as part of the NORMAL project, outside the submitted work.

## Supporting citations

References (160,161,162,163,164,165,166,167,168,169,170,171,172,173,174,175,176) appear in the supporting material.

## Supporting material

Document S1. Tables S1–S5

Table S6. Summary of articles linking to data sets or supplemental files

Document S2. Article plus supporting material
