# Supplementary material for: FAIR data for optical tweezers experiments
Source: Biophys J. 2025 Mar 12;124(8):1255–72. doi: 10.1016/j.bpj.2025.03.005 (PMC12044397; doi:10.1016/j.bpj.2025.03.005)
Supplement: Document S1. Tables S1–S5 [file mmc1.pdf]

**Biophysical Journal, Volume *124***

# Supplemental information

# FAIR data for optical tweezers experiments

**Matthew T.J. Halma, Sowmiyaa Kumar, Jan van Eck, Sanne Abeln, Alexander Gates, and Gijs J.L. Wuite**

**Supplementary Information**

Table S1: An overview of the FAIR principles and their criteria. Adapted from (1).

| | |
|---|---|
| Findable | |
| F1 | (Meta)data are assigned a globally unique and persistent identifier. |
| F2 | Data are described with rich metadata. |
| F3 | Metadata clearly and explicitly include the identifier of the data they describe. |
| F4 | (Meta)data are registered or indexed in a searchable resource. |
| Accessible | |
| A1 | (Meta)data are retrievable by their identifier using a standardized communications protocol. |
| A1.1 | The protocol is open, free, and universally implementable. |
| A1.2 | The protocol allows for an authentication and authorization procedure, where necessary. |
| A2 | Metadata are accessible, even when the data are no longer available. |
| Interoperable | |
| I1 | (Meta)data use a formal, accessible, shared, and broadly applicable language for knowledge representation. |
| I2 | (Meta)data use vocabularies that follow FAIR principles. |
| I3 | (Meta)data include qualified references to other (meta)data. |
| Reusable | |
| R1 | (Meta)data are richly described with a plurality of accurate and relevant attributes. |
| R1.1 | (Meta)data are released with a clear and accessible data usage license. |
| R1.2 | (Meta)data are associated with detailed provenance. |
| R1.3 | (Meta)data meet domain-relevant community standards. |

In a first search the data availability of the ten high-impact papers based on citations with a publication date between 2015 and 2020 was analyzed. The Mendeley database was searched for the term “optical tweezers”, and the results were sorted by number of citations. As citation counts differ slightly between different sources, the articles are not in perfect descending order by Google Scholar citation number. Six papers did not provide any information about their datasets, two papers stated to contact the authors, and one paper provided representative data traces as figures in the supplementary information, and another

article included complete data for each figure. This preliminary search already shows the gap concerning the FAIR principles (Table S2).

Table S2. A search of ten high-impact optical Tweezers publications

| Article | Google Scholar Citations (Accessed November 21, 2024) | Supplementary Materials? | Raw dataset? |
|---|---|---|---|
| Neupane et al., 2016. *Science* (2) | 246 | Yes | No |
| Das et al., 2015. *Proceedings of the National Academy of Sciences* (3) | 252 | Yes | No |
| Goldman et al., 2015. *Science* (4) | 258 | Yes | No |
| Kilchherr et al., 2016. *Science* (5) | 223 | Yes | No |
| Comstock et al., 2015. *Science* (6) | 212 | Yes | No |
| Mashaghi et al., 2016. *Nature* (7) | 205 | Yes | Data available upon request |
| Feng et al., 2017. Proceedings of the National Academy of Sciences (8) | 194 | Yes | No, some representative data traces as figures in SI |

| | | | |
|---|---|---|---|
| Brouwer et al., 2016. *Nature* (9) | 184 | Yes | Yes |
| Nicholas et al., 2015. *Proceedings of the National Academy of Sciences* (10) | 123 | Yes | No |
| Wasserman et al., 2019. *Cell* (11) | 132 | Yes | Data available upon request |

Of the articles, one had a dataset which was available on the site. We evaluate using the FAIR Criteria (Table S1). We include the FAIR analysis of the only result from the search to include an associated raw dataset(9).

Table S3. A FAIR Assessment of the article from Table S2 with a linked dataset based on the criteria in Table S1.

| Brouwer, I., G. Sitters, A. Candelli, S.J. Heerema, I. Heller, A.J. Melo de, H. Zhang, D. Normanno, M. Modesti, E.J.G. Peterman, et al. 2016. Sliding sleeves of XRCC4–XLF bridge DNA and connect fragments of broken DNA. *Nature*. 535:566–569. | | |
|---|---|---|
| F1 | Partial | Article DOI but not dataset DOI |
| F2 | Sufficient | Metadata in supplementary data files and methods section. |
| F3 | Sufficient | Metadata described with official names in article text |
| F4 | Insufficient | This can be considered a limitation of the field. There are not search engines besides standard article search engines that can easily find this dataset. |
| A1.1 | Sufficient | No access restrictions |
| A1.2 | Sufficient | No access restrictions |
| A2 | Partial | Metadata are in article text, may still be viewable even if dataset becomes unavailable |
| I1 | Partial | The files are stored as excel |

| | | |
|---|---|---|
| | | files which are accessible, but people without MS Excel may face issues |
| I2 | Sufficient | Units are included in files. |
| I3 | Partial | Formal vocabulary used, but entities not linked. |
| R1.1 | Partial | No data usage license on page. User will have to make a RightsLink Request. |
| R1.2 | Sufficient | Detailed provenance information in methods section of article. |
| R1.3 | Not applicable | No widely accepted OT community standards |

A different search strategy was implemented to find papers with associated datasets. In the second search for data, we located ten papers from journals with open data policies, with publication dates from 2019 to 2020. This date range is chosen because open data practices in several journals were adopted in 2018, making 2019 the first full year of open data policies. This search with term "Optical Tweezers" in the OpenAlex database (12) for the ten most-cited experimental papers with a biological subject published 2019 or 2020, resulted in a total of two usable force-extension datasets.

Table S4. A search of ten recent optical tweezers publications in journals with an open data policy.

| Article | Google Scholar Citations (Accessed November 21, 2024) | Supplementary Materials? | Raw dataset? |
|---|---|---|---|
| Newton et al. 2019. *Nature Structural and Molecular Biology* (13) | 137 | Yes | Data available upon request |
| Avellaneda et al., 2020. *Nature*(14) | 107 | Yes | Data available upon request |

| | | | |
|---|---|---|---|
| Liu et al. 2019 *Molecular Cell* (15) | 94 | Yes | Data available upon request |
| Chen et al. 2019 *eLife* (16) | 81 | Yes | Link to Dryad repository with raw data broken |
| Dewulf et al. 2019. *Nature Communications* (17) | 69 | Yes | Data available upon request |
| Shu et al. 2020. *Proceedings of the National Academy of Sciences.* (18) | 68 | Yes | No |
| Rao et al., 2019. *Nature Communications* (19) | 50 | Yes | Yes |
| Huis in't Veld et al. 2019. *eLife.* (20) | 51 | Yes | No, includes detailed derived data |
| Patrick et al., 2020. *Nature Chemical Biology* (21) | 47 | Yes | Data available upon request |
| Halma et al. 2019. *Proceedings of the National Academy of Sciences* (22) | 51 | Yes | No |

Table S5. A FAIR Assessment of the article with a linked dataset from Table S4 based on the criteria in Table S1.

| Rao, L., F. Berger, M.P. Nicholas, and A. Gennerich. 2019. Molecular mechanism of cytoplasmic dynein tension sensing. *Nature communications*. 10:1–17. | | |
|---|---|---|
| F1 | Partial | Immutable article DOI, but not separate dataset DOI |
| F2 | Sufficient | Detailed metadata in supplementary data file. |
| F3 | Sufficient | Metadata described with official names |
| F4 | Insufficient | This can be considered a limitation of the field. There are not search engines besides standard article search engines that can easily find this dataset. |
| A1.1 | Sufficient | No access restrictions |
| A1.2 | Sufficient | No access restrictions |
| A2 | Partial | Metadata are in article text |
| I1 | Partial | The files are stored as excel files which are accessible, but people without MS Excel may face issues |
| I2 | Sufficient | Units are included in files. |
| I3 | Partial | Formal vocabulary used, but entities not linked. |
| R1 | Sufficient | Provenance information is detailed enough to allow replication. |
| R1.1 | Sufficient | Creative Commons Attribution 4.0 International License. |
| R1.2 | Sufficient | Detailed provenance information in methods section of the article. |
| R1.3 | Not applicable | No widely accepted OT community standards |

**References**

1. Jacobsen, A., R. de Miranda Azevedo, N. Juty, D. Batista, S. Coles, R. Cornet, M. Courtot, M. Crosas, M. Dumontier, C.T. Evelo, et al. 2020. FAIR Principles: Interpretations and Implementation Considerations. *Data Intell*. 2:10–29.

2. Neupane, K., D.A.N. Foster, D.R. Dee, H. Yu, F. Wang, and M.T. Woodside. 2016. Direct observation of transition paths during the folding of proteins and nucleic acids. *Science*. 352:239–242.

3. Das, D.K., Y. Feng, R.J. Mallis, X. Li, D.B. Keskin, R.E. Hussey, S.K. Brady, J.-H. Wang, G. Wagner, E.L. Reinherz, et al. 2015. Force-dependent transition in the T-cell receptor β-subunit allosterically regulates peptide discrimination and pMHC bond lifetime. *Proc. Natl. Acad. Sci. U.S.A.* 112:1517–1522.

4. Goldman, D.H., C.M. Kaiser, A. Milin, M. Righini, I. Tinoco, and C. Bustamante. 2015. Ribosome. Mechanical force releases nascent chain-mediated ribosome arrest in vitro and in vivo. *Science*. 348:457–460.

5. Kilchherr, F., C. Wachauf, B. Pelz, M. Rief, M. Zacharias, and H. Dietz. 2016. Single-molecule dissection of stacking forces in DNA. *Science*. 353:aaf5508.

6. Comstock, M.J., K.D. Whitley, H. Jia, J. Sokoloski, T.M. Lohman, T. Ha, and Y.R. Chemla. 2015. Direct observation of structure-function relationship in a nucleic acid -processing enzyme. *Science*. 348:352–354.

7. Mashaghi, A., S. Bezrukavnikov, D.P. Minde, A.S. Wentink, R. Kityk, B. Zachmann-Brand, M.P. Mayer, G. Kramer, B. Bukau, and S.J. Tans. 2016. Alternative modes of client binding enable functional plasticity of Hsp70. *Nature*. 539:448–451.

8. Feng, Y., K.N. Brazin, E. Kobayashi, R.J. Mallis, E.L. Reinherz, and M.J. Lang. 2017. Mechanosensing drives acuity of αβ T-cell recognition. . *Proc. Natl. Acad. Sci. U.S.A.* 114:E8204–E8213.

9. Brouwer, I., G. Sitters, A. Candelli, S.J. Heerema, I. Heller, A.J. Melo de, H. Zhang, D. Normanno, M. Modesti, E.J.G. Peterman, et al. 2016. Sliding sleeves of XRCC4–XLF bridge DNA and connect fragments of broken DNA. *Nature*. 535:566–569.

10. Nicholas, M.P., F. Berger, L. Rao, S. Brenner, C. Cho, and A. Gennerich. 2015. Cytoplasmic dynein regulates its attachment to microtubules via nucleotide state-switched mechanosensing at multiple AAA domains. . *Proc. Natl. Acad. Sci. U.S.A.* 112:6371–6376.

11. Wasserman, M.R., G.D. Schauer, M.E. O'Donnell, and S. Liu. 2019. Replication Fork Activation Is Enabled by a Single-Stranded DNA Gate in CMG Helicase. *Cell*. 178:600-611.e16.

12. Priem, J., H. Piwowar, and R. Orr. 2022. OpenAlex: A fully-open index of scholarly works, authors, venues, institutions, and concepts. ArXiv, arXiv:2205.01833, http://arxiv.org/abs/2205.01833 (preprint posted 17 Jun 2022).

13. Newton, M.D., B.J. Taylor, R.P.C. Driessen, L. Roos, N. Cvetesic, S. Allyjaun, B. Lenhard, M.E. Cuomo, and D.S. Rueda. 2019. DNA stretching induces Cas9 off-target activity. *Nat. Struct. Mol. Biol*. 26:185–192.

14. Avellaneda, M.J., K.B. Franke, V. Sunderlikova, B. Bukau, A. Mogk, and S.J. Tans. 2020. Processive extrusion of polypeptide loops by a Hsp100 disaggregase. *Nature*. 578:317–320.

15. Liu, K., K. Maciuba, and C.M. Kaiser. 2019. The Ribosome Cooperates with a Chaperone to Guide Multi-domain Protein Folding. *Mol. Cell*. 74:310-319.e7.

16. Chen, Z., R. Gabizon, A.I. Brown, A. Lee, A. Song, C. Díaz-Celis, C.D. Kaplan, E.F. Koslover, T. Yao, and C. Bustamante. 2019. High-resolution and high-accuracy topographic and transcriptional maps of the nucleosome barrier. *eLife*. 8:e48281.

17. Dewulf, M., D.V. Köster, B. Sinha, C. Viaris de Lesegno, V. Chambon, A. Bigot, M. Bensalah, E. Negroni, N. Tardif, et al. 2019. Dystrophy-associated caveolin-3 mutations reveal that caveolae couple IL6/STAT3 signaling with mechanosensing in human muscle cells. *Nat. Commun*. 10:1974.

18. Shu, T., H. Jin, J.E. Rothman, and Y. Zhang. 2020. Munc13-1 MUN domain and Munc18-1 cooperatively chaperone SNARE assembly through a tetrameric complex. *Proc. Natl. Acad. Sci. U.S.A.* 117:1036–1041.

19. Rao, L., F. Berger, M.P. Nicholas, and A. Gennerich. 2019. Molecular mechanism of cytoplasmic dynein tension sensing. *Nat. Commun*. 10:1–17.

20. Veld, P.J.H. in ’t, V.A. Volkov, I.D. Stender, A. Musacchio, and M. Dogterom. 2019. Molecular determinants of the Ska-Ndc80 interaction and their influence on microtubule tracking and force-coupling. *eLife*.

21. Patrick, E.M., J.D. Slivka, B. Payne, M.J. Comstock, and J.C. Schmidt. 2020. Observation of processive telomerase catalysis using high-resolution optical tweezers. *Nat. Chem. Biol*. 1–9.

22. Halma, M.T.J., D.B. Ritchie, T.R. Cappellano, K. Neupane, and M.T. Woodside. 2019. Complex dynamics under tension in a high-efficiency frameshift stimulatory structure. *PNAS*. 116:19500–19505.
